# Bio-based polylactic acid labware as a sustainable alternative for microbial cultivation in life science laboratories

**DOI:** 10.1016/j.heliyon.2024.e39846

**Published:** 2024-10-26

**Authors:** Jennie O Loughlin, Bevin Herward, Dylan Doherty, Purabi Bhagabati, Susan M. Kelleher, Samantha Fahy, Brian Freeland, Keith D. Rochfort, Jennifer Gaughran

**Affiliations:** a School of Physical Sciences, Dublin City University, D9 Dublin, Ireland; b School of Chemical Sciences, Dublin City University, D9 Dublin, Ireland; c Office of the Chief Operations Officer, Dublin City University, D9 Dublin, Ireland; d School of Biotechnology, Dublin City University, D9 Dublin, Ireland; e Life Sciences Institute, Dublin City University, D9 Dublin, Ireland

**Keywords:** Polylactic acid, Cell viability, Optical sensors, Sustainability, Circular bioeconomy

## Abstract

Single-use plastics (SUPs) in life science laboratories account for approximately 5.5 million tonnes of waste per year globally. Of SUPs used in life science laboratories, Petri dishes, centrifuge tubes, and inoculation loops are some of the most common. In order to reduce the reliance on petrochemical-based SUPs in the life science research laboratory and minimize the negative environmental impacts associated with SUPs, this research investigates the applicability of polylactic acid (PLA) in single-use labware as a replacement for petrochemical-based plastics. PLA is one of the most well-studied biodegradable plastics that can be produced from sustainable resources. Commercially available PLA was used to 3D print a select range of labware to test the suitability of PLA-based material for routine microbiology work. An injection moulded PLA-based Petri dish was also designed and produced, for increased optical clarity. The biocompatibility was tested against Gram-negative (*Escherichia coli*) and Gram-positive (*Staphylococcus epidermidis*) strains of bacteria. The PLA-based labware did not negatively impact the cell growth, viability, and metabolic activity of the bacterial cultures. The injection moulded PLA Petri dish showed a reduced colony forming unit count for the Gram-negative *E. coli* strain compared to the polystyrene Petri dish, ∼1.5 × 10^9^ CFU/mL and ∼3.0 × 10^9^ CFU/mL respectively, during late-exponential growth. The colony counts were, however, in the same order of magnitude. This observed difference may be due to the internal environment inside the Petri dish, hence the internal O_2_ concentration, humidity, and temperature during bacterial growth were investigated. This work demonstrates, for the first time, a full successful workflow of bacterial growth using a sustainable bioplastic, providing a pathway to reducing the environmental impacts of SUPs in life science laboratories.

## Graphical abstract

**Figure:**
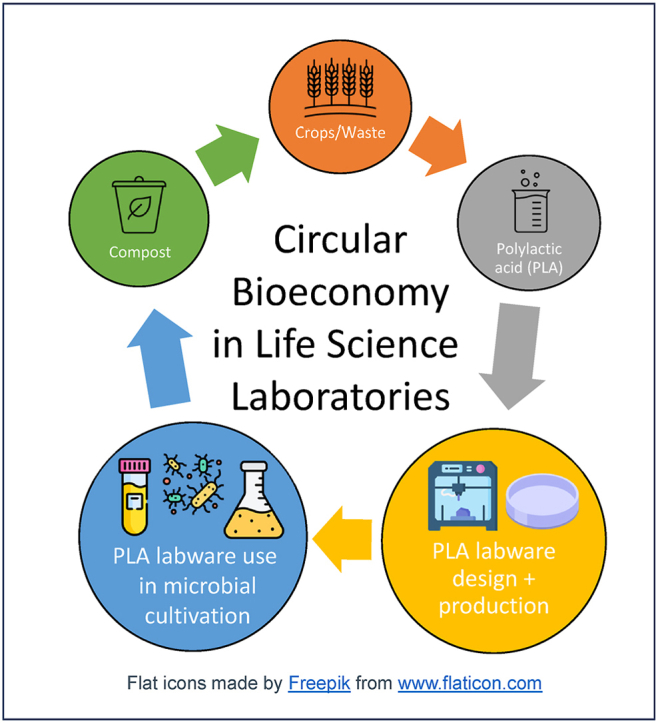

## 1 Introduction

In the past 50 years, global plastic production has grown 20-fold [1]. In 2019, ∼360 million tonnes of petrochemical-based plastics was produced globally [2]. Although the COVID-19 pandemic led to a slight reduction in global plastic production, an increase in solid plastic waste was observed, due in part to the use of single-use plastics (SUPs) in Personal Protective Equipment (PPE) [3,4]. SUPs are disposed of either by recycling, incineration or landfilling, with landfilling being the most common [5]. 79 % of global plastic waste ended up in landfill in 2015 [6]. Macro- and microplastic release from landfills lead to environmental pollution and negative health effects [7,8]. There is a growing push to develop more sustainable alternatives to replace the currently used petrochemical SUPs. The EU has committed to becoming climate neutral and to establishing a circular economy by 2050, as laid out in the ‘Green Deal’ [9]. A major part of this plan aims to reduce the reliance on petrochemical SUPs. This will be accomplished by banning SUPs where appropriate and pivoting from petrochemical, non-biodegradable plastics to bio-based, biodegradable plastics.

**Fig. 1:**
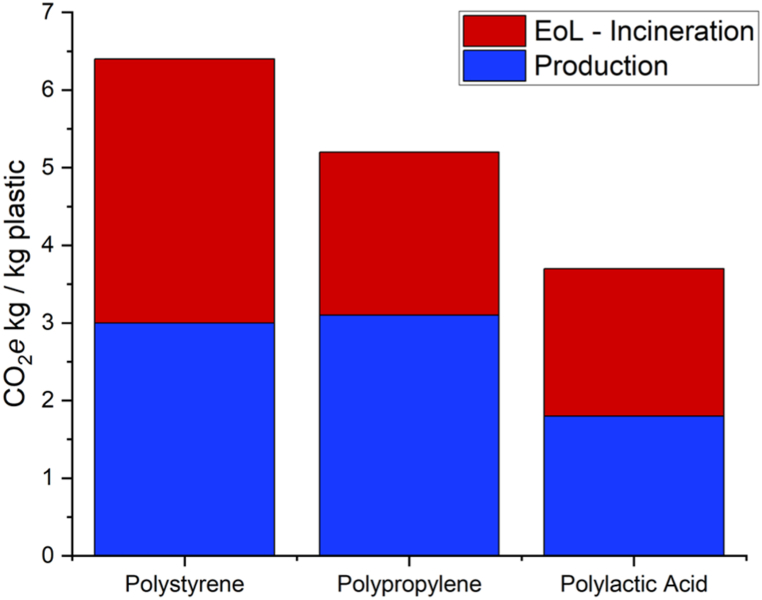
The estimated carbon footprint of polystyrene (PS), polypropylene (PP), and polylactic acid (PLA) production and incineration EoL option only [[19], [20], [21], [22], [23]].

Over 99 % of plastics are derived from fossil fuels [10] but there is a trend towards the development of bio-based, biodegradable plastics in recent years, influenced by worldwide government incentives to decrease reliance on fossil fuels [11]. It is estimated that global bioplastics production will increase from 2.18 million tonnes in 2023 to 7.43 million tonnes by 2028 [12]. Bio-based plastics, such as bio-polyethylene (bio-PE) are produced from renewable, biomass sources while biodegradable plastics, such as polycaprolactone (PCL), can be degraded by living organisms to produce CO_2_ and H_2_O [13]. Certain plastics such as polylactic acid (PLA) are both bio-based and biodegradable, allowing for a transition towards a circular economy, decreased greenhouse gas (GHG) emissions, and carbon sequestration during plastic production [14,15]. Lactic acid, the starting material for PLA, can be produced directly from feedstocks [16] or from various industrial waste streams [17,18]. Life cycle assessments have been conducted on the environmental impacts of petrochemical and bio-based plastics production and end-of-life options, showing lower overall CO_2_ emissions for bio-based plastics such as PLA (Fig. 1).

PLA has become a leading bioplastic, holding the largest market share in 2023 [12]. The relative ease-of-use during processing is a major advantage of PLA. It can be used in extrusion, biaxial stretching, and injection moulding processes [[24], [25], [26]]. The mechanical, thermal, and chemical properties of PLA make it a suitable replacement for petrochemical-based plastics in a variety of applications [27]. It is used in the healthcare industry in sutures and implants due to its biocompatibility and degradability resulting in non-toxic products [26,28]. Research is ongoing regarding the use of PLA as a drug delivery vehicle [29,30]. It is widely used in the food packaging industry due to its high transparency, chemical resistance to fats and oils, and ultraviolet light barrier properties [13,31]. Moreover, PLA can be produced from bacterial fermentation processes, and is biodegradable under industrial composting conditions, positioning it as a strong candidate for use in circular bio-economies [32]. The use of PLA as a replacement material for single-use plastic labware in the life sciences laboratory has previously been reviewed [33]. The focus on PLA as the bioplastic of choice was initially based on its manufacturing capacity compared to other biodegradable bioplastics. In 2023, the global production capacity of PLA was 31 %, compared to the nearest competitor, polyhydroxyalkanoates (PHA), with a global production capacity of 4.8 % [12]. The ease of production of PLA components for rapid prototyping using fused deposition modelling (FDM) technology has been established [34]. PLA's mechanical properties are comparable to certain plastics commonly used in life science laboratories, such as PS, although it is more brittle than other commonly used plastics such as PP and PC [33].

Although PLA is a biodegradable material, biodegradation will only occur in industrial composting conditions (≥58 °C) within the time frame indicated by international standards e.g. ISO 14885-1 [35]. Waste plastics from labware are biologically contaminated in general, hence incineration is the preferred EoL. The carbon savings associated with replacing petrochemical plastics with bioplastics such as PLA mainly arise from the plastics production, as indicated in Fig. 1. These developments show that PLA could be a viable replacement for traditional petrochemical-based plastics in areas where SUPs still play a significant role.

**Fig. 2:**
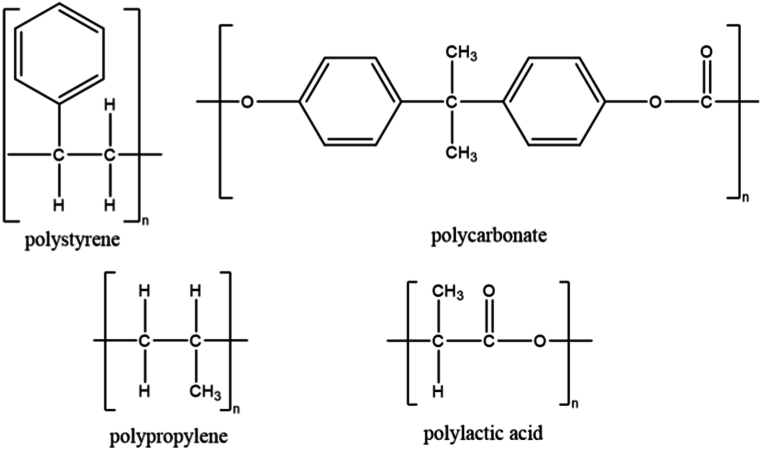
Monomeric structures of common petrochemical polymers and bio-based polylactic acid.

It has been estimated that 5.5 million tonnes of laboratory SUP waste was produced globally in 2014 [36]. SUP is convenient in the life sciences laboratory as the products are pre-sterilized and can be easily disposed of once contaminated. In this way, SUP is cost effective in small-scale through to large-scale bioprocesses [37]. Commonly used petrochemical-based plastic labware is made from a variety of polymers, depending on the final use. These polymers include polystyrene (PS), polycarbonate (PC), and polypropylene (PP) (Fig. 2) and their properties have been detailed previously [33,38]. The microbiology laboratory has essential functions in society, from clinical point-of-care to food production and thus significantly contributes to the laboratory SUP waste each year [[39], [40], [41]].

The thermal properties of PLA set limitations on the range of end uses for this bioplastic. PLA has a heat deflection temperature of 55 °C, compared to 75 °C for PS and 65 °C for PP [42]. Physical ageing of PLA has been studied, resulting in a more brittle material. This aging process occurs more rapidly in humid conditions and in direct sunlight [43,44]. The molecular weight and glass transition temperature of PLA decreases during this process, while the percentage crystallinity increases. These limitations are considered when designing the 3D printed labware, sterilizing the labware before microbial culturing, storing the labware, and determining the conditions for microbial cultivation. Molecular and structural characteristics of PLA, such as molecular weight and percentage crystallinity, affect the mechanical and thermal properties of the material. These parameters for the 3D printed and injection moulded PLA material will hence be outlined. This study will investigate the utility of PLA-based labware as a replacement for traditional, petrochemical-based labware.

Both Gram-negative and Gram-positive bacterial strains were examined, and indices for cell growth, metabolic activity, and viability of cultures grown solely using PLA-based labware were benchmarked against cultures grown using traditional labware. PLA products for mammalian cell cultivation currently exist on the market and we have previously demonstrated that 3D printed PLA Petri dishes meet all the required standards for use in laboratories [45,46]. Select PLA labware has previously been designed and 3D printed [47]. To our knowledge, this is the first published study of routine bacterial cultivation using only PLA-based plastics throughout the full workflow as a replacement for petrochemical-based plastics.

## 2 Materials and methods

### 2.1 Materials

All chemicals and reagents were purchased from Fisher Scientific™. Bacterial strains *Escherichia coli* (*E. coli*, 500173, DH5α) and *Staphylococcus epidermidis* (*S. epidermidis*, ATCC 12228) were purchased from Thermo Fisher Scientific.

Three PS Petri dish international brands were chosen to compare to PLA Petri dishes (Table 1).

**Table 1:** Codes and external dimensions of the three commercially available PS Petri dishes chosen for this study.

PS Petri dish code	External Dimensions	
	Diameter (mm)	Height (mm)
PS 1	94	16
PS 2	90	16
PS 3	100	20

The Ultimaker S3 from Ultimaker (Utrecht, the Netherlands), a FDM printer was used to 3D print the select labware. Ultimaker Transparent PLA filament was used, acquired from Inspire3D (Rathnew, Co. Wicklow, Ireland). The filament had a diameter of 2.85 ± 0.10 mm and was extruded using the Ultimaker 0.4 AA print core.

PLA Petri dishes (93/18 mm) were injection moulded at Smallwares Injection Moulding, Dundalk, Ireland. NatureWorksLLC Ingeo™ 3251D PLA and TotalEnergies Corbion Luminy® L130 PLA were used. Physical and thermal properties of the three PLA polymers after 3D printing or injection moulding are given in Table 2.

**Table 2:** Physical properties of PLA after processing.

PLA source	Weight Average Molecular Weight (M_W,_ kDa)	Number Average Molecular Weight (M_n,_ kDa)	Polydispersity Index (PDI = M_W_/M_n_)	% Crystallinity	Optical Purity (% L)
Inspire3D Ultimaker, Transparent	134	58	2.3	3.5	–
NatureWorksLLC Ingeo™ 3251D	106	64	1.7	7.3	∼98.6 [48]
TotalEnergies Corbion Luminy® L130	120	52	2.3	14.9	>99 [49]

### 2.2 Methods

#### 2.2.1 3D printing of PLA labware

Print settings reported by Doherty et al. were used to prepare the labware used in this work. A print speed of 15 mm/s, a layer height of 0.04 mm and a print temperature of 200 °C were used as reported [46]. The 3D printed Petri dish had a thickness of 1 mm for the base and the lid. PLA will replace PS in this instance and 1 mm thickness ensured the Petri dish complied with ISO 24998, while also maintaining a degree of optical transparency. The 3D printed conical flask and centrifuge tube had a thickness of 1 mm and 2 mm, respectively. This design was to account for the increased brittleness of PLA compared to PC and PP.

#### 2.2.2 Materials testing

Gel permeation chromatography (GPC, Agilent 1260Infinity II-MDS System) was used to determine the molecular weights and weight distributions of the PLA material after 3D printing or injection moulding. The GPC was fitted with a guard column followed by two PLgel 5 μm MIXED-D 300 × 7.5 mm columns and simultaneously fitted with a refractive index detector (RID) and viscometer (VS). For system calibration, narrow linear PSstandards for column calibration (EasiVial PS-M range of nominal Mp 162–400,000 Da) were used. Samples were dissolved in THF, 0.1 % (w/v) and left overnight before being filtered over 0.22 μm nylon syringe filters prior to analysis. Measurements were taken at 30 °C, using THF as the eluent with a flow rate of 1 mL/min. Agilent GPC/SEC Software was used to carry out the analysis. Data provided in Table 2.

Differential scanning calorimetry (DSC 4000 System, PerkinElmer) was used to determine the percentage crystallinity of the 3D printed and injection moulded PLA material. Crystallinity effects the mechanical and thermal properties of PLA, hence the importance of determining this parameter before application of the PLA labware. Samples (∼10 mg) were enclosed in aluminium pans and crimped. The programming of all test samples follow the standard heating-cooling-heating cycle. In the first heating cycle, the samples were heated from −10 °C to 220 °C at a rate of 20 °C/min to erase any unwanted thermal history in the polymers during processing, which was followed by the cooling cycle down to −10 °C at the same rate. In the second heating cycle, the samples were then heated again from −10 °C to 220 °C at a rate of 20 °C/min. For the calculation of percentage crystallinity, Equation (1) was used where the data presented is collected from the second heating cycle only.

Equation 1 $$X_{c}=\frac{\Delta h_{m}-\Delta h_{\text{cc}}\;}{\Delta h_{m}^{0}}\times 100\;\%$$

The specific enthalpy of melting (J/g, Δh_m_) and specific enthalpy of cold crystallization (J/g, Δh_cc_) were obtained from second heating cycle. The theoretical enthalpy of melting of 100 % crystalline PLA (Δh_m_^0^) is taken to be 93 J/g [50,51]. Data is provided in Table 2.

#### 2.2.3 Biological compatibility of PLA labware

**Fig. 3:**
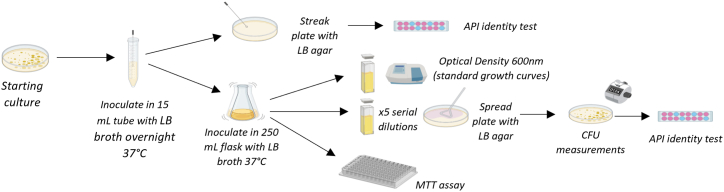
The bacterial cultivation workflow in this study. Initially, each PLA-based labware under investigation was substituted in for the petrochemical equivalent, keeping all other petrochemical labware the same. The bacterial growth, metabolic activity and purity was monitored and benchmarked against the bacterial cultivation using only petrochemical labware. Image generated using BioRender.

The utility of each piece of labware (Petri dish, 15 mL centrifuge tube, 250 mL shake flask, inoculation loop, L-shaped spreader, cuvettes) was firstly investigated by replacing the petrochemical equivalent with the PLA-based labware in a typical workflow for bacterial cultivation (Fig. 3). Workflows with petrochemical labware only were run in unison, as a control study. Each experiment was carried out in triplicate.

Starting colonies of *E. coli* or *S. epidermidis* on an LB (lysogeny broth) agar Petri dish were collected and inoculated into 7–8 mL LB in a 15 mL tube. This inoculation was placed at 37 °C, 250 RPM overnight (∼18 h). A sample of the overnight culture was diluted 1:100 and 100 μL of this dilution was spread on fresh LB agar plates, which were placed at 37 °C overnight. The identity of randomly selected colonies on these plates were confirmed using API 20E or API Staph test strips and reagents.

A sample of the overnight culture was taken and placed in 100 mL fresh LB in a 250 mL flask, such that the optical density at 600 nm (OD_600_) was 0.01–0.02. The 250 mL flask was placed at 37 °C, 250 RPM. An OD_600_ reading was taken every hour over 10 h and the growth curves of the bacterial culture were plotted.

The viability of the cell culture was determined by collecting a sample of the culture during the early, mid, and late exponential growth stages. Serial dilutions were made using fresh LB (1 × 10^1^ - 1 × 10^6^) and 100 μL of each dilution was spread on LB agar plates. The plates were placed at 37 °C overnight. Colony forming units (CFU) were counted on the plates the following day, excluding plates with <50 or >300 colonies.

The metabolic activity of the cell culture was determined by the MTT reduction assay [52,53]. (3-(4,5-dimethylthiazol-2-yl)-2,5-diphenyltetrazolium bromide (MTT) was dissolved in deionised water (5 g/L) and preheated at 37 °C. Fresh LB broth was also preheated at 37 °C, along with 1.5 mL centrifuge tubes. At early, mid, and late exponential growth, samples were taken from the growth culture and diluted to an OD_600_ of 0.1 using the preheated LB. 200 μL of this dilution was placed in the preheated 1.5 mL centrifuge tube and 20 μL of the MTT solution was added (final MTT concentration: 0.45 g/L). The sample was heated at 37 °C for 20 min. The samples were centrifuged at 2000 g × 2 min. The supernatant was removed, taking care to not disturb the pellet. The pellet was dissolved in 1 mL DMSO. The solution was then transferred to a glass vial. A further 1.5 mL DMSO was used to wash the centrifuge tube and all washings were added to the solution in the glass vial. Absorbance readings at 550 nm of the solution were taken, using DMSO as the blank. An MTT Reduction Unit (MRU) per mL of growth culture was then calculated. Negative controls of no MTT (20 μL of deionised water only) and no cell culture (200 μL of preheated LB only) were used.

#### 2.2.4 Petri dish internal environment

Temperature, humidity, and O_2_ concentration was investigated within the environment of the closed Petri dish during colony growth of *E. coli* at 37 °C on 25 mL LB agar. The Multicomp Pro 83–17985 Temp/Humidity Sensors were placed inside the PS 1 Petri dish, inside the PLA IM 2 Petri dish, and in the external environment. An Arduino Uno microcontroller board and Arduino Software (IDE) were used to collect the temperature and humidity data. PreSens oxygen sensor spots SP-PSt3-NAU were used to measure the O_2_ in the airspace inside the Petri dish. An OXY-1 SMA meter and PreSens measurement studio 2 were used to collect and display the data. Air saturation of O_2_ (%) data was collected at 5 min intervals during incubation and it took up to 50 data points for oxygen sensor calibration to occur at 37 °C. 100 μL of mid-exponential growth *E. coli* culture was diluted (x10^−4^) in LB and spread on the Petri dish and the temperature/humidity or O_2_ concentration was monitored overnight at 37 °C.

#### 2.2.5 Statistical analysis

Statistical analysis was performed with OriginPro2023. Paired sample *t*-tests were carried out to determine significance differences between means. The null hypothesis was set as the mean of viability measurements of bacteria grown with petrochemical labware remains unchanged when bacteria are grown with PLA labware. Significance level was set at p < 0.05. Average colony diameters were obtained using ImageJ (https://imagej.net/ij/)

## 3 Results

### 3.1 Biological compatibility of PLA labware

**Fig. 4:**
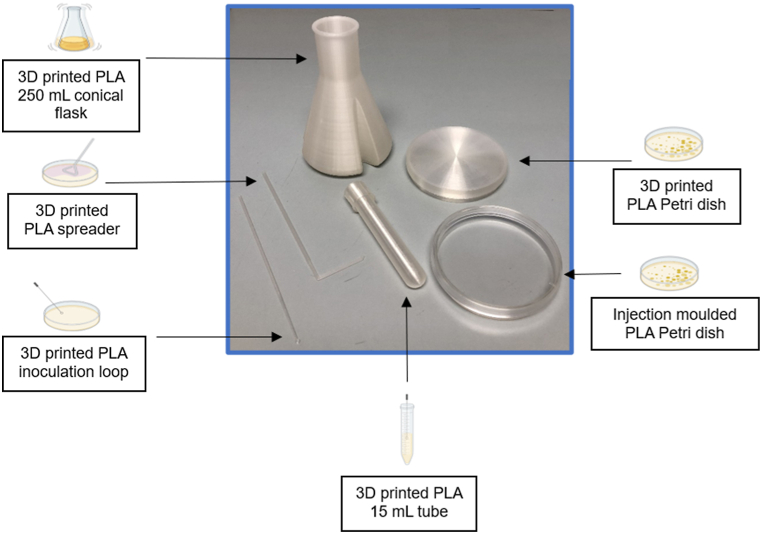
Selection of 3D printed PLA-based labware and injection moulded PLA-based Petri dish. Image generated using BioRender.

PLA-based 250 mL conical flasks, 15 mL tubes, 90 mm Petri dishes, inoculation loops, and L-shaped spreaders were 3D printed (Fig. 4). This labware, along with the PLA-based injection moulded Petri dishes were packaged and sterilized by vaporized hydrogen peroxide (VHP, 30 % H2O2 concentration, 65 °C, ambient pressure, 6–9 h). The sterility of PLA plastics after this sterilization technique has previously been examined [46].

Although the 3D printed PLA Petri dish facilitated bacterial growth on agar, comparable to commercially available petrochemical-based Petri dishes, the optical clarity of the 3D printed PLA Petri dish was not adequate for controlled viewing of bacterial colonies, even after polishing (using Akasel A/S polishing kit). The Petri dish was injection moulded using high heat and high flow grade PLA, for improved optical clarity (Fig. S1).

**Fig. 5:**
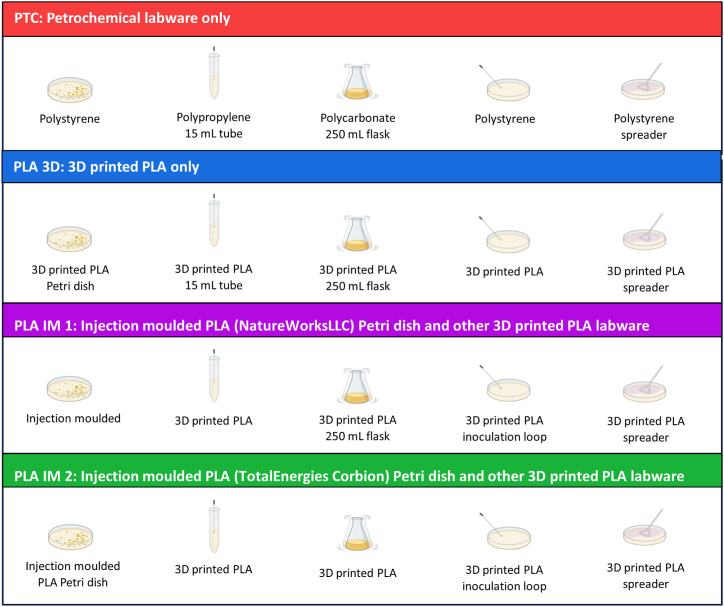
Labware used in indicated workflows (denoted by PTC, PLA 3D, PLA IM 1, PLA IM 2) to benchmark PLA labware against petrochemical labware. A) The PTC workflow contained only standard petrochemical labware. B) The PLA 3D workflow contained only 3D printed PLA labware. C) The PLA IM 1 workflow contained only PLA labware, with the Petri dishes injection moulded from NatureWorksLLC PLA and all other components 3D printed. D) The PLA IM 2 workflow contained only PLA labware, with the Petri dishes injection moulded from TotalEnergies Corbion PLA and all other components 3D printed. Image generated using BioRender.

The Gram-negative strain DH5alpha *Escherichia coli* was chosen as this is a common, versatile strain used in teaching, research and industry for gene cloning and cell proliferation [54]. *Staphylococcus epidermidis* was chosen as the Gram-positive bacterial strain, to investigate whether the outer bacterial cell structure affected the growth and viability of the culture when in contact with PLA. The optimum growth temperature for both strains is 37 °C. This is below the heat deflection temperature (∼55 °C) and glass transition temperature (∼60 °C) of PLA [33]. Morphological changes are observed in PLA at and above these temperatures. Therefore, mesophilic bacteria were chosen for this study. This illustrates a limitation of neat PLA-based labware, in that it is not suitable for thermophilic bacterial cultivation above 55 °C. The utility of each piece of PLA labware was independently tested in the cultivation of *E. coli* and *S. epidermidis* by substituting the petrochemical alternative with the selected PLA labware, using traditional, petrochemical labware in all other aspects of the workflow. The bacterial strains were then cultivated using only PLA-based labware where possible and the viability of the cultures were compared to the cultures grown using only petrochemical labware. There were four main workflows at this stage (Fig. 5). The results detailed here describe these four workflows, unless otherwise specified.

**Fig. 6:**
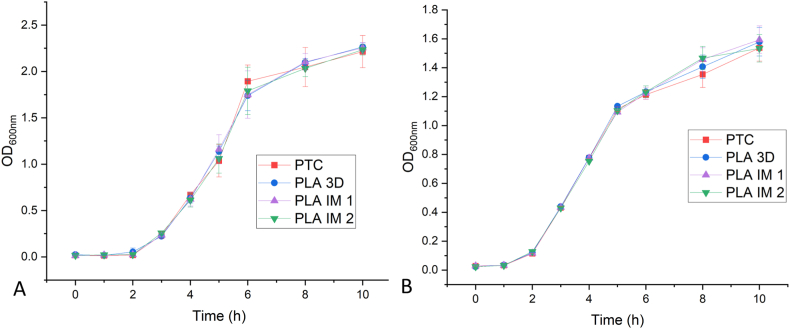
Growth curves (A: *E. coli* and B: *S. epidermidis*) for all four workflows (PTC, PLA 3D, PLA IM 1 and PLA IM 2) obtained by measuring the optical density at 600 nm over 10 h, in LB medium at 37 °C. Data is expressed as mean ± SD (n = 3).

Cell viability was first examined, for all four workflows, by following the growth of the strains in LB at 37 °C for 10 h (Fig. 6). There was no statistical difference in cell growth using 3D printed PLA-based labware when compared to petrochemical plastics. *E. coli* cells had a doubling time of 18 min while *S. epidermidis* had a doubling time of 32 min using both plastics (Table 3). Three different cuvettes were also used during the PTC workflow when culturing *E. coli*. Commercially available PS, 3D printed Ultimaker PLA, and injection moulded NatureWorks PLA cuvettes were compared when taking OD_600nm_ measurements. This data is given in Fig. S2.

**Table 3:** Growth rates (μ) and doubling times (dT) for the bacterial strains.

	*E. coli*		S. epidermidis	
	Growth rate (μ,/h)	Doubling time (dT, min)	Growth rate (μ,/h)	Doubling time (dT, min)
PTC	2.303 ± 0.02	18	1.319 ± 0.08	32
PLA 3D	2.305 ± 0.02	18	1.281 ± 0.07	32
PLA IM 1	2.307 ± 0.03	18	1.302 ± 0.08	32
PLA IM 2	2.305 ± 0.02	18	1.310 ± 0.06	32

**Fig. 7:**
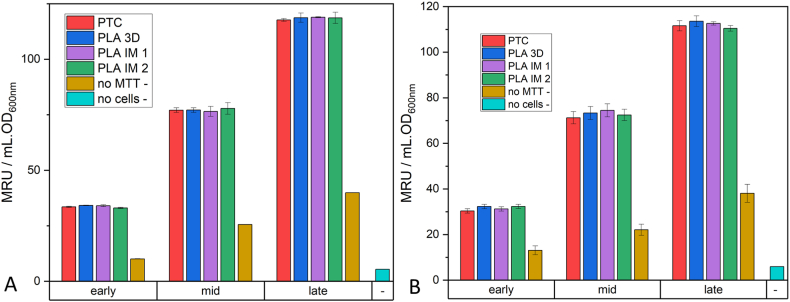
MRU values (MTT Reduction Units, A: *E. coli* and B: *S. epidermidis*) obtained from absorbance at 550 nm when cells (early-, mid-, and late-exponential growth) were treated with MTT (0.45 g/L final concentration), incubated at 37 °C for 15 min and resuspended in DMSO. Data is expressed as mean ± SD (n = 3).

The viability of the cell cultures was further investigated using the MTT assay. This colorimetric assay is widely used to determine the metabolic and enzymatic activities of bacterial cultures [55]. The metabolic activity of the cultures was investigated during the early-, mid-, and late-exponential stages of growth (Fig. 7). The time after inoculation of overnight culture into fresh LB for these 3 exponential stages for each bacterial strain is given in Table S1. There was no statistical difference (p > 0.05) in the MRU values calculated for the cultures grown in each of the four workflows. Negative controls of cell culture samples with no MTT at different bacterial growth stages and a negative control with no cell culture were used to assess the background absorbance at 550 nm associated with this assay.

#### 3.1.1 Petri dish CFU variability

**Fig. 8:**
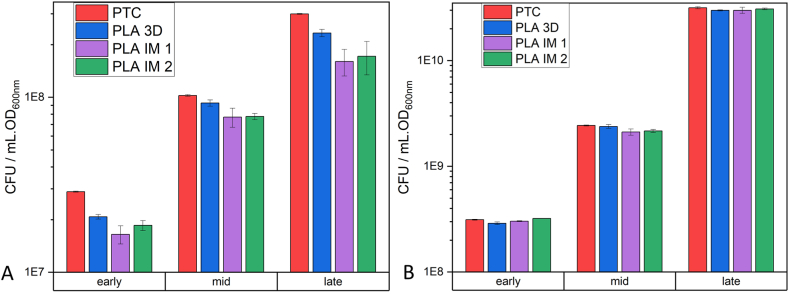
CFU values (A: *E. coli* and B: *S. epidermidis*) obtained from the colony count on four different Petri dishes when incubated overnight at 37 °C. Samples were collected and diluted during early-, mid-, and late-exponential growth. Data is expressed as mean ± SD (n = 3).

Colony-forming unit (CFU) assays are widely used and are seen as the ‘gold standard’ for determining cell viability in microbiology [56,57]. Like the MTT assay, cell samples were taken during the early-, mid-, and late-exponential growth stages (Fig. 8) of each workflow and spread on the Petri dish. Both strains are facultative anaerobic, therefore the spread plate technique was initially used with 100 μL of the serial dilutions spread on the surface of the LB agar. The identification of randomly selected colonies was verified using the appropriate API testing kit (API20E for *E. coli* and API STAPH for *S. epidermidis*). All selected colonies were identified as either *E. coli* or *S. epidermidis*, proving the sterility of the PLA labware after VHP sterilization.

The mean CFU values for *S. epidermidis* were statistically the same across all Petri dishes. The average diameter of colonies grown on all dishes was 0.62 ± 0.01 mm. The mean CFU values for *E. coli* dilutions spread on the PLA IM 1 and PLA IM 2 Petri dishes were statistically lower than for the PS 1 Petri dishes (p < 0.05), although the CFU per mL of culture was calculated to be in the same order of magnitude (10^7^ to 10^8^ cells/mL, depending on the stage of growth) (Fig. S3). The average diameter of colonies grown on PS 1 Petri dishes was 1.72 ± 0.40 mm. The average diameter of colonies grown on PLA IM 1 Petri dishes was 2.52 ± 0.21 mm, while on PLA IM 2 Petri dishes it was 2.68 ± 0.27 mm. All data is given in Table S2. The difference between the two bacterial strains could be due to the different growth rates and patterns and area covered by the colonies (3–4 mm for *E. coli* [58], 1–2 mm for *S. epidermidis* [59]).

To determine if the difference in *E. coli* CFUs was due to the PLA polymer leaching into the agar (poured into the Petri dish at 45 °C), the pour plate technique was used, where the serial dilution of *E. coli* culture was mixed with molten LB agar (15 mL). This agar was allowed to solidify and then a further 10 mL of molten LB agar was poured over it. The *E. coli* colonies were allowed to grow at 37 °C, 18 h in this anaerobic environment [60]. Under these conditions, the CFU counts for PS 1 Petri dishes and PLA IM 1 and PLA IM 2 Petri dishes were statistically the same. The viability of the colonies grown on the PLA Petri dishes was investigated by growing the colonies in fresh LB at 37 °C using petrochemical labware (PTC workflow, Fig. 5) and comparing the growth to the colonies grown on the PS 1 Petri dish. The MTT assay was also carried out on the cultures. No statistical differences were observed in the cell cultures from colonies collected from PS 1 Petri dishes vs. PLA IM 1 and PLA IM 2 Petri dishes (Fig. S4).

**Fig. 9:**
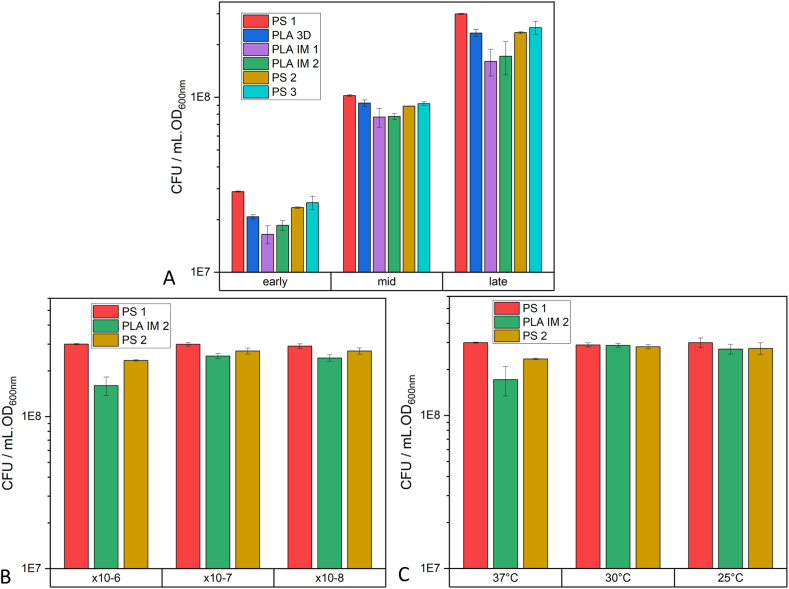
A) CFU values of *E. coli* obtained from the colony count on Petri dishes when incubated overnight at 37 °C. Samples were collected during the early-, mid-, and late-exponential growth stages. B) CFU values of *E. coli* obtained from the colony count on Petri dishes at different dilutions when incubated overnight at 37 °C. C) CFU values of *E. coli* obtained from the colony count on Petri dishes at when incubated at 37 °C (16 h), 30 °C (2 days), or 25 °C (4 days). Samples were collected during the mid-exponential growth stage and diluted. Data is expressed as mean ± SD (n = 3).

When different brands of PS Petri dishes were investigated (PS 2 and PS 3) statistically different CFUs were calculated (p > 0.5), with serial dilutions taken from the same *E. coli* culture (Fig. 9A). The concentration of the serial dilution spread on the Petri dish influenced the calculated CFU. When the *E. coli* culture was diluted so that ∼300 colonies grew on the PS 1 Petri dish, the same sample led to ∼165 colonies on the PLA IM 1 and PLA IM 2 Petri dishes. The same diluted sample led to ∼240 colonies on the PS 2 and PS 3 Petri dishes. However, if the *E. coli* culture was diluted so that 50–150 colonies grew on the PS 1 Petri dish, the same number of colonies grew on the PLA IM 2 and PS 2 Petri dishes (Fig. 9B, Fig. S5).

*E. coli* was grown on PS 1, PS 2, and PLA IM 2 Petri dishes at 30 °C (for 2 days) and 25 °C (for 4 days) to determine if the growth rate of *E. coli* impacted the CFU counts. When incubated at 30 °C and 25 °C, the CFU counts on the three Petri dishes tested were statistically the same (Fig. 9C). The average diameter of colonies grown on the Petri dishes were all within the range 2.03 ± 0.83 mm. Average CFU counts, and diameter measurements are given in Table S2.

In summary, a significant difference was observed in the CFU count of *E. coli* grown on the surface of the LB agar at 37 °C when using PLA Petri dishes compared to the PS 1 dish. The average diameters of the resulting colonies grown on the PS vs. PLA dishes were also different, with larger colonies growing the on the PLA IM 2 and PLA IM 1 dishes. No significant differences were observed between *S. epidermidis* CFU counts and colony size on the different Petri dishes. The viability of the *E. coli* colonies grown on PS 1 and all three PLA Petri dishes was verified by cultivating the colonies in LB in petrochemical labware following PTC workflow (Fig. 5). There was no significant difference in cell viability and metabolism of cultures with starting colony from the PS 1 Petri dish vs. all PLA Petri dishes. Other commercially available PS Petri dishes were investigated, with slightly different dimensions to the PS 1 Petri dishes. Significantly different *E. coli* CFU counts were obtained under the same conditions. Altering the concentration of the cell culture spread on the agar of the Petri dish or the temperature of growth impacted the final CFU count of *E. coli* grown on PLA IM 2 and PS 2 Petri dishes.

### 3.2 Petri dish internal environment

**Fig. 10:**
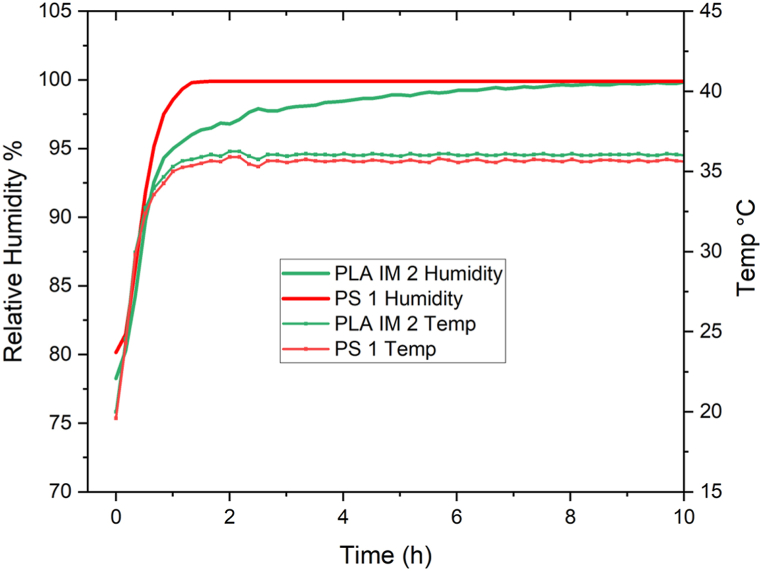
The RH (%) and temperature (°C) of PS 1 and PLA IM 2 Petri dishes with 25 mL LB agar was recorded (n = 3) in an incubator set at 37 °C. An external temp/humidity sensor was placed in the incubator, showing a steady recording of 36.5 ± 0.5 °C and 20.4 ± 0.4 RH %.

Taking all if this into account, it was postulated that the difference in calculated CFU was due to the environment inside the Petri dish (temperature, humidity, O_2_ concentration), impacted by the design of the dish. Studies were conducted to determine the temperature and humidity inside the PS 1 Petri dish and the PLA IM 2 Petri dish at 37 °C with 25 mL sterile LB agar. Multicomp Pro 83–17985 temperature and humidity sensors placed inside the Petri dishes with LB agar recorded the temperature (°C) and humidity (relative humidity, RH %) inside the dishes when placed in an incubator set at 37 °C for 24 h. An external temp/humidity sensor was placed in the incubator, showing a steady recording of 36.5 ± 0.5 °C and 20.4 ± 0.4 RH %. The results of the temperature and humidity recordings are given in Fig. 10. The temperatures within the PS 1 and PLA IM 2 Petri dishes are within the same range (36.5 ± 0.5 °C), comparable to the external environment. The humidity inside the PS 1 Petri dish reaches saturation (99.9 % RH) after ∼ 1.5 h at 37 °C. The humidity inside the PLA IM 2 Petri dish reaches saturation (99.9 % RH) after ∼ 8.5 h at 37 °C. This may be due to this larger volume of headspace in the PLA IM 2 Petri dish (62.85 cm^3^) compared to the PS 1 dish (60.60 cm^3^) as the internal height of the injection moulded PLA Petri dish design is 1.1 ± 0.2 mm greater than the PS 1 Petri dish (dimensions given in Table S3).

**Fig. 11:**
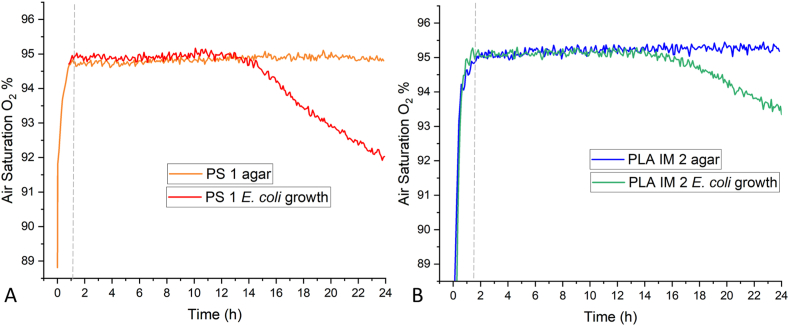
A) The O_2_ air saturation (%) in the PS 1 Petri dish with agar (orange line) and agar and *E. coli* growth (yellow line) at 37 °C, 24 h. Air saturation of O_2_ begins to deplete after ∼13 h incubation during *E. coli* growth. B) The O_2_ air saturation (%) in the PLA IM 2 Petri dish with agar (blue line) and agar and *E. coli* growth (purple line) at 37 °C, 24 h. Air saturation of O_2_ begins to deplete after ∼14 h incubation during *E. coli* growth. Grey dashed line indicates the time taken for sensor calibration in the 37 °C incubator to occur. All data is expressed as mean (n = 3).

The available O_2_ in the headspace of the PS 1 Petri dish and PLA IM 2 Petri dish was investigated using PreSens optical oxygen sensors fixed to the centre of the lid of each Petri dish. The non-invasive optical sensors have previously been used to record the O_2_ concentration (%) and O_2_ uptake rate (OUR, mmol/L/h) of *E. coli* in liquid medium [61,62]. O_2_ consumption rates of both *E. coli* and *S. epidermidis* have been investigated in liquid medium under various conditions and using various techniques [[63], [64], [65]]. In this study, the O_2_ concentration of the air was recorded as air saturation (%). The Petri dishes with 25 mL LB agar were investigated initially, followed by *E. coli* growth on 25 mL LB agar at 37 °C for 24 h (Fig. 11). Average data (n = 3) is provided in Table 4. During incubation, O_2_ depletion in the PS 1 Petri dish begins 13 h after initial incubation, while O_2_ begins to deplete after 14 h in the PLA IM 2 Petri dish. The rate of O_2_ depletion in the PS 1 dish is 0.29 %/h compared to 0.18 %/h in the PLA IM 2 Petri dish. At atmospheric pressure (1 atm) and 37 °C, the mass of O_2_ in the headspace is calculated to be 14.48 mg in the PS 1 Petri dish and 15.77 mg in the PLA IM 2 Petri dish. O_2_ depleted at a rate of 0.042 mg/h in the PS 1 dish and 0.027 mg/h in the PLA IM 2 dish.

**Table 4:** The steady state air saturation of O_2_ (%) in PS 1 and PLA IM 2 Petri dishes with 25 mL LB agar is given. Beginning of O_2_ depletion during *E. coli* growth is indicated, as well as the rate of O_2_ depletion up to 24 h incubation.

	PS 1 + 25 mL LB agar	PLA IM 2 + 25 mL LB agar
Average O_2_ Air Saturation %	94.81 ± 0.15	95.18 ± 0.18
Start of O_2_ depletion at 37 °C incubation during bacterial growth	∼13 h	∼14 h
Rate of O_2_ depletion (% air saturation) up to 24 h incubation %/h	0.288 ± 0.003 R^2^ = 0.988	0.179 ± 0.003 R^2^ = 0.961
Headspace (mL) above 25 mL agar	60.60	65.97
Mass (mg) of O_2_ in headspace above 25 mL agar	14.48	15.77
Rate of O_2_ depletion (mg) up to 24 h incubation mg/h	0.042 R^2^ = 0.988	0.028 R^2^ = 0.962

**Fig. 12:**
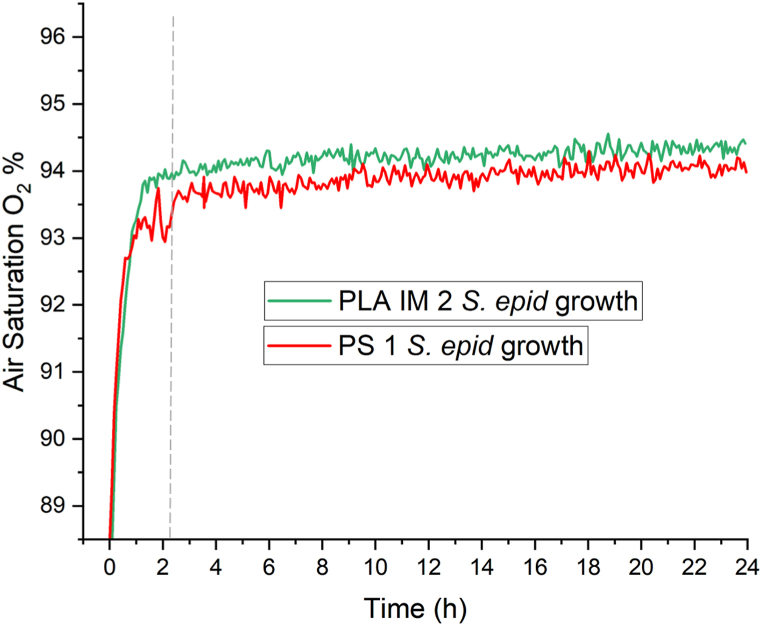
A) The O_2_ air saturation (%) in the PS 1 Petri dish with agar and *S. epidermidis* growth (yellow line) at 37 °C, 24 h and the O_2_ air saturation (%) in the PLA IM 2 Petri dish with agar and *S. epidermidis* growth (purple line) at 37 °C, 24 h. Grey dashed line indicates the time taken for sensor calibration in the 37 °C incubator to occur. All data is expressed as mean (n = 3).

The O_2_ air saturation % was investigated during *S. epidermidis* growth in the PS 1 and PLA IM 2 Petri dishes, to compare the O_2_ depletion to *E. coli* growth. The was no observable depletion of O_2_ in either Petri dish during *S. epidermidis* growth on 25 mL LB agar at 37 °C (Fig. 12).

## 4 Discussion

The difference in CFU counts between the PLA and PS 1 Petri dishes for *E. coli* when incubated at 37 °C (Fig. 8A) - the initial experimental conditions - led to various routes of investigation. This difference occurred when using the ‘spread-plate’ technique, where bacterial culture grows on the surface of the agar. The ‘pour-plate’ technique was hence used to grow *E. coli* colonies under identical conditions. No difference in CFU counts between the PLA and PS dishes were observed in this study. Thus, leaching of lactic acid or PLA oligomers into the molten agar was ruled out. pH testing of agar poured at 45 °C also did not indicate a decrease in pH due to lactic acid in the agar. No CFU count differences were observed for the other bacterial strain S. epidermidis cultivated on the PLA and PS 1 dishes (Fig. 8B). *E. coli* was then grown on two other commercially available PS dishes (PS 2 and PS 3) and statistically significant differences in CFU counts were observed among all three PS dishes, although they were all in the same order of magnitude. This finding, along with the finding that when the incubation temperature is lower than 37 °C (30 °C and 25 °C), the *E. coli* CFU count is statistically the same among all PS and PLA Petri dishes, led to the hypotheses that the internal environment inside the Petri dish (O_2_ availability, humidity) could be affecting the rate and pattern of growth of *E. coli* colonies. At 37 °C, the doubling time of *E. coli* is 20 min in liquid culture. Given this fast growth rate, these environmental factors may play a more significant role than when *E. coli* is incubated at a lower temperature or for a slower growing bacterial strain (doubling time for S. epidermidis ≈35 min at 37 °C). O_2_ concentration in the head space of PLA IM 2 and PS 1 Petri dish was monitored over the *E. coli* incubation period at 37 °C. The PLA dish did not show a lower initial O_2_ concentration. In fact, the initial O_2_ concentration was maintained in the PLA Petri dish for longer than in the PS Petri dish, possible due to the increased headspace volume of the PLA Petri dish, due to the design dimensions. The humidity within the Petri dishes was also monitored during *E. coli* incubation at 37 °C. The humidity within the PS dish reached saturation after ∼1.5 h of incubation, while saturation in the PLA dish was only reached after ∼8.5 h. O_2_ availability, culture vessels, and incubation conditions have been studied on bacterial growth and viability, mainly in liquid broth [[66], [67], [68]]. The effect of humidity on bacterial growth has also been studied, with reduced humidity decreasing bacterial growth [69]. In a morphological study of the impact of RH (%) on Gram-positive (B. cereus) and Gram-negative (*E. coli*), the Gram-negative strain was found to be more susceptible to a reduction in RH, compared to the Gram-positive strain [70]. These small differences of the internal environment inside the Petri dish may lead to changes in the growth patterns of the *E. coli* strain, as observed in the final CFU counts. The increased *E. coli* colony diameter observed in the PLA IM 2 Petri dish can be explained by the recent finding of the spatial distribution and colony size correlation of *E. coli* on agar. It was shown that colony size was proportional to geometric area [71]. Therefore, fewer colonies distributed on the same area of agar may lead to increased individual colony size. This difference in time of saturation is due to the difference in headspace within the Petri dish. The monitoring of O_2_ and humidity in culture vessels for liquid media is commonplace in microbial cultivation. To the authors knowledge, there are no studies that monitor these environmental conditions in a culture vessel for microbial growth on solid media (i.e. agar). This study is the first of its kind in highlighting the importance of component design for microbial growth on solid media.

PLA manufacturing is an evolving technology. PLA is currently used in short term food packaging and in the medical industry for drug delivery systems and bioabsorbable medical implants, for example [26,72]. PLA is an important polymer in the relatively new area of 4D printing technologies, with recent studies investigating the shape-memory properties of PLA FDM materials for biomedical applications [73,74]. These applications underline the biocompatibility of neat PLA material. A limited range of PLA single use labware is currently on the market, for mammalian cell culture [45,75]. A recent study highlighted the use of sterile 3D printed PLA shake flasks for mammalian cell cultivation [76]. The findings detailed here build upon the understanding of PLA as a suitable biobased plastic for use in life science laboratories, investigating 3D printed and injection moulded PLA labware for bacterial cultivation in routine microbiology workflows, at mesophilic conditions. Although GHG emission reductions are achieved when petrochemical-based plastics are replace with PLA [77], the limitations of neat PLA have been outlined. Neat PLA can be used as described here for single use, but it is not suitable for reuse. PLA labware could not be sterilized via autoclave or UV radiation without loss of structural integrity or mechanical properties [46]. There have been recent studies to improve the thermal properties of PLA through biobased composites and blends [[78], [79], [80]], including polyamide blends and nucleating agents such as N,N′-ethylenebis(12-hydroxystearamide) (EBHS). An autoclavable, 3D printed bioreactor vessel has been produced using temperature stable, commercially available PLA filament [81]. Future studies investigating the use of labware produced using tailored PLA blends must be conducted.

## 5 Conclusion

In this study, the application of PLA-based labware to replace common petrochemical plastics in routine microbiological techniques was investigated. The growth and viability of a Gram-negative (*E. coli*) and a Gram-positive (*S. epidermidis*) bacterial strain, cultivated using 3D printed and injection moulded PLA-based labware (250 mL shake flasks, 15 mL test tubes, Petri dishes, inoculation loops, spreaders), was compared to the growth and viability of each strain using traditional petrochemical-based, commercially available plastic labware. The growth and viability of both strains were not negatively impacted by cultivation using PLA labware, as shown in the growth curves and calculated growth rates and using the MTT viability assay. The CFU counts of both strains cultivated in 250 mL PLA shake flasks were comparable to the CFU counts of the bacterial strains cultivated in 250 mL PC shake flasks, when diluted culture was spread on PS 1 Petri dishes. However, there was a statistically significant difference in the CFU count of culture incubated on PS 1 Petri dishes compared to culture incubated on PLA dishes. Differences in CFU counts for *E. coli* were also observed between various commercially available PS Petri dishes. This indicates that CFU measurements are affected by the design of the Petri dish itself. It was shown that the differences in the internal environment (head space, RH %) may affect the growth patterns of certain bacterial strains on agar.

Further studies are required to determine the long-term storage effects on PLA labware. Physical ageing affecting structural integrity and thermal stability of PLA is well known. Therefore, microbial cultivation using PLA labware that has been stored for ≥1 year should be studied to determine what, if any, impact the change in PLA's mechanical and thermal properties during aging has on PLA-based labware performance. Different storage conditions (dry/humid, sunlight/darkness, low temperatures (4 °C, −20 °C)) should also be considered. Biopolymer blends and nucleating agents such as EBHS will also be investigated during the production of PLA-based labware. These blends and nucleating agents have been shown in the literature to improve the thermal stability and to reduce the brittleness of the material. It may not only improve the properties of the PLA material but could result in reusable biobased labware. The petrochemical plastic PC is used in plastic conical flasks for microbial culture incubation. These conical flasks are sterilized by autoclave after each experiment and reused. PLA, as 3D printed or injection moulded in this study, could not be autoclaved or UV radiated without significant loss in structural integrity. Development of a biobased plastic blend that could withstand routine sterilization and could hence be reused would provide a further improvement in terms on carbon footprint reduction. Nevertheless, this study confirms for the first time the applicability of PLA-based labware as a feasible alternative to petrochemical-based labware for microbial cultivation. This is an important step towards reducing the carbon footprint of SUP production.

## CRediT authorship contribution statement

**Jennie O Loughlin:** Writing – original draft, Visualization, Methodology, Investigation, Formal analysis, Data curation. **Bevin Herward:** Investigation, Data curation. **Dylan Doherty:** Methodology, Investigation. **Purabi Bhagabati:** Writing – review & editing, Formal analysis. **Susan M. Kelleher:** Funding acquisition. **Samantha Fahy:** Funding acquisition. **Brian Freeland:** Funding acquisition. **Keith D. Rochfort:** Writing – review & editing, Supervision, Funding acquisition. **Jennifer Gaughran:** Writing – review & editing, Supervision, Funding acquisition.

## Data availability

Raw data will be made available upon request.

## Declaration of competing interest

The authors declare that they have no known competing financial interests or personal relationships that could have appeared to influence the work reported in this paper.
