# Supplementary material for: Bio-based polylactic acid labware as a sustainable alternative for microbial cultivation in life science laboratories
Source: Heliyon. 2024 Oct 26;10(21):e39846. doi: 10.1016/j.heliyon.2024.e39846 (PMC11558637; doi:10.1016/j.heliyon.2024.e39846)
Supplement: Multimedia component 1 [file mmc1.docx]

Bio-based Polylactic Acid (PLA) Labware as a Sustainable Alternative for Microbial Cultivation in Life Science Laboratories


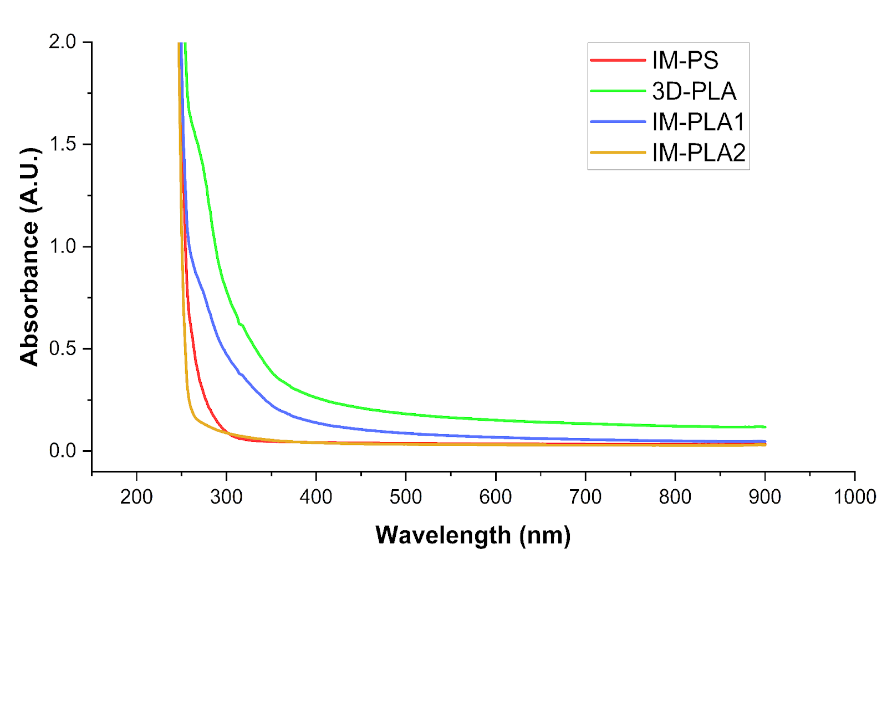
Supplementary Materials

PS 1 PLA 3D PLA IM 1 PLA IM 2


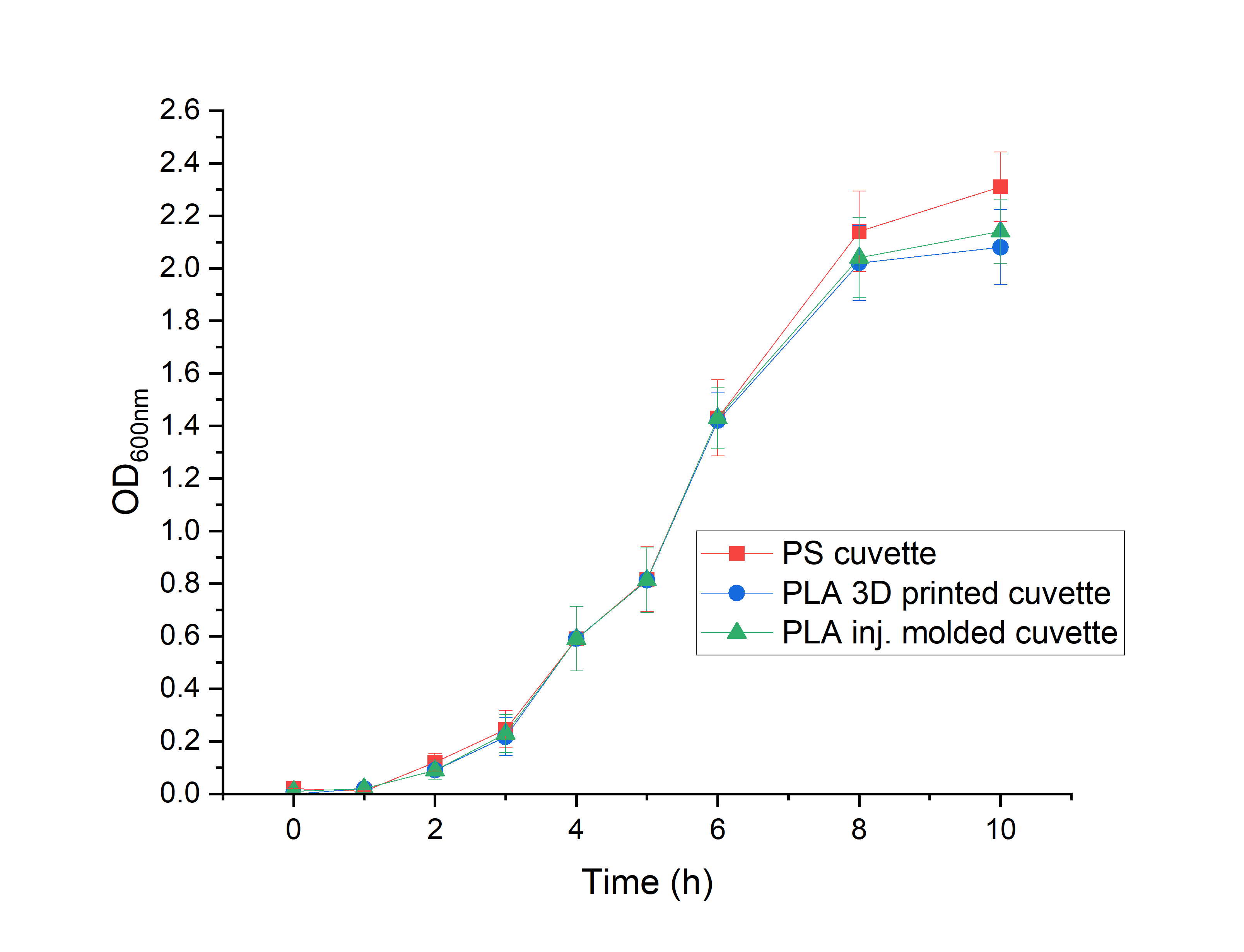
 Figure S1. Absorbance measurements of polystyrene (PS) and PLA Petri dishes from 230 nm - 900 nm, measured using a Tecan Infinite Pro MPlex microplate reader.

Figure S2. Growth curves of *E. coli* in LB medium at 37°C during workflow PTC, obtained by measuring the optical density at 600 nm over 10 h with 3 different cuvettes as indicated. Data is expressed as mean + SD (n = 3).


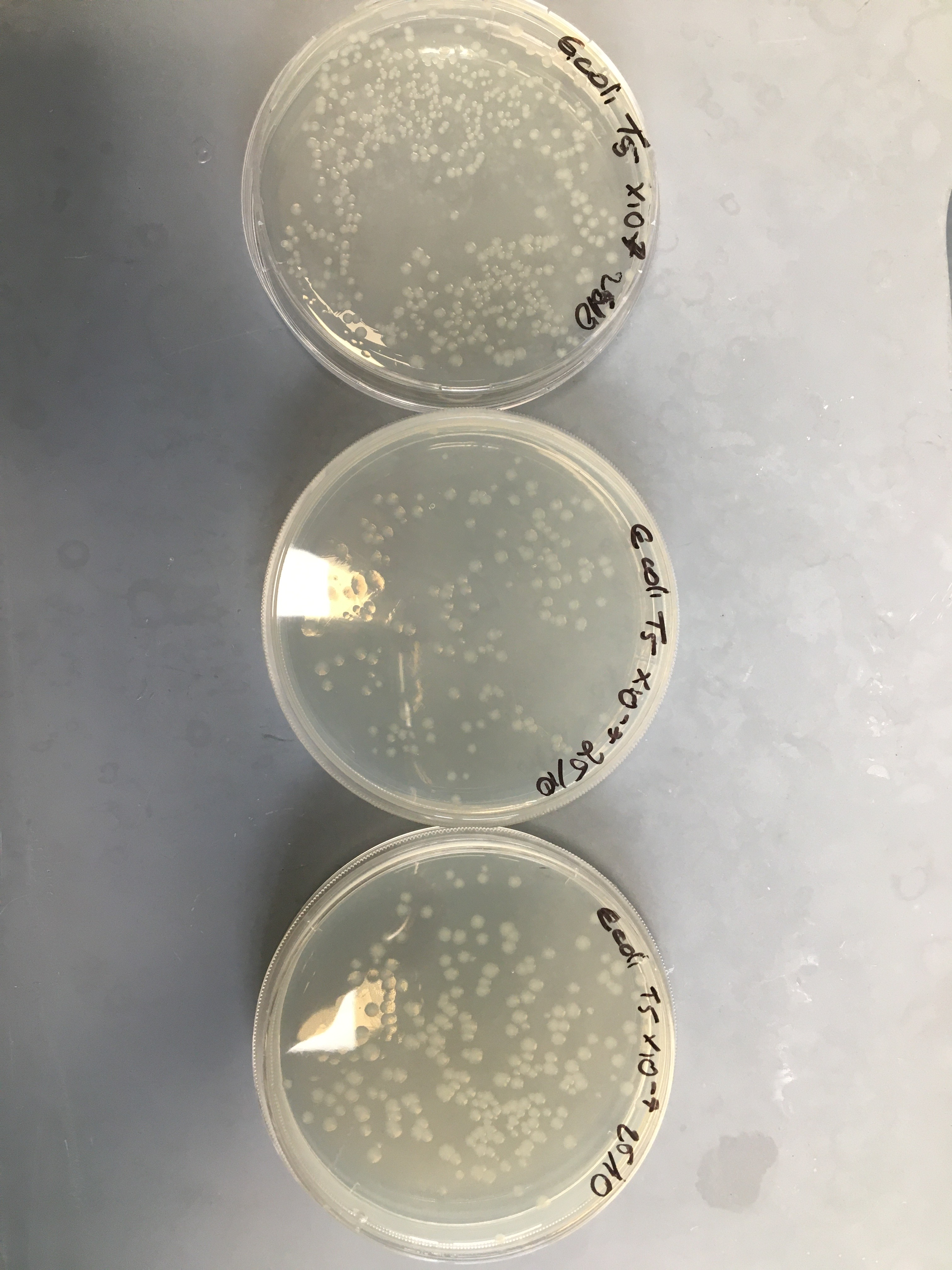


3

2

1

A


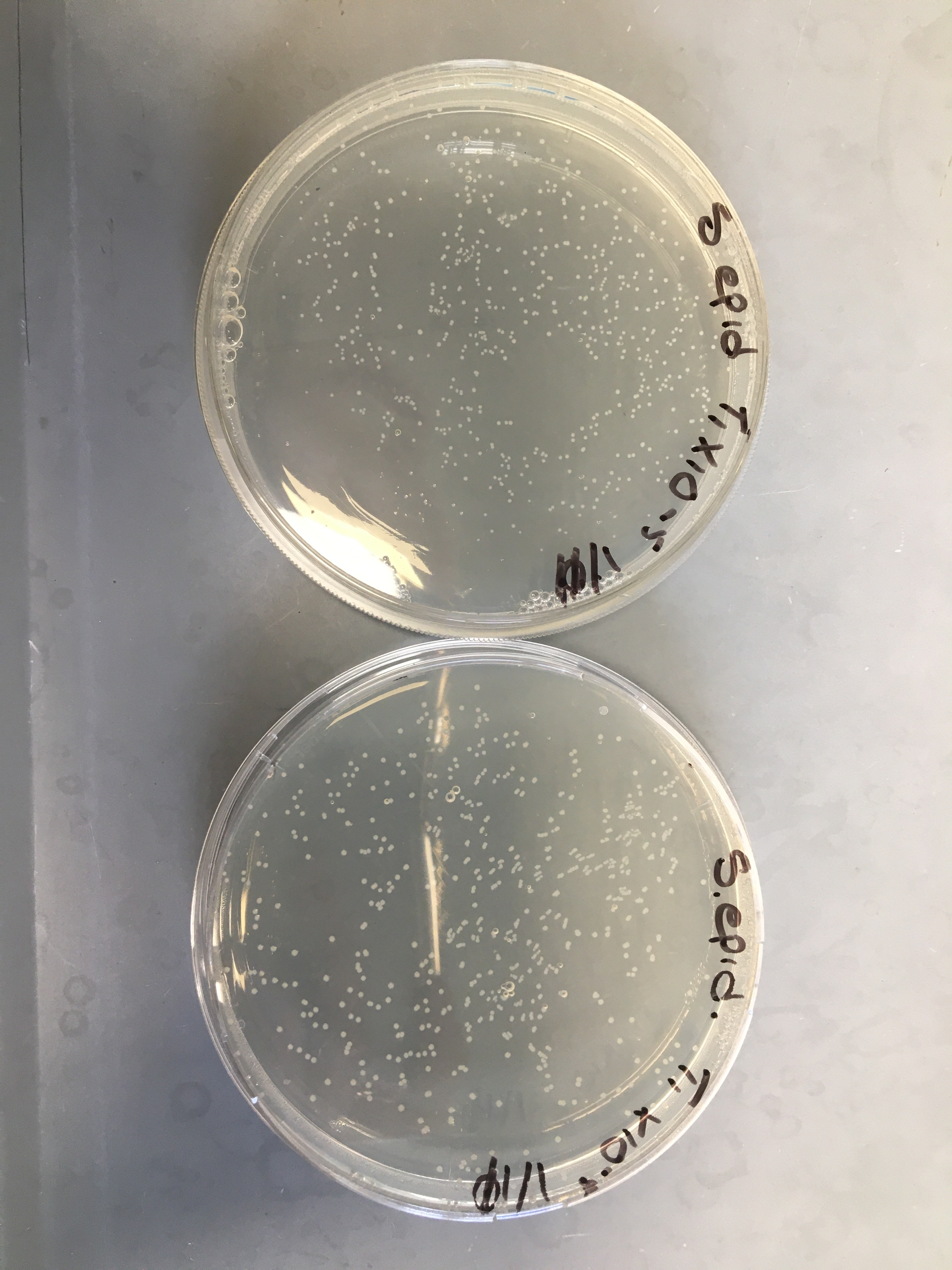


2

1

B

Figure S3. A) CFU counts of *E. coli* during late-exponential stage on 1) PS 1 2) PLA IM 1 and 3) PLA IM 2 Petri dishes. B) CFU counts of *S. epidermidis* during late-exponential stage on 1) PS 1 and 2) PLA IM 2 Petri dishes.


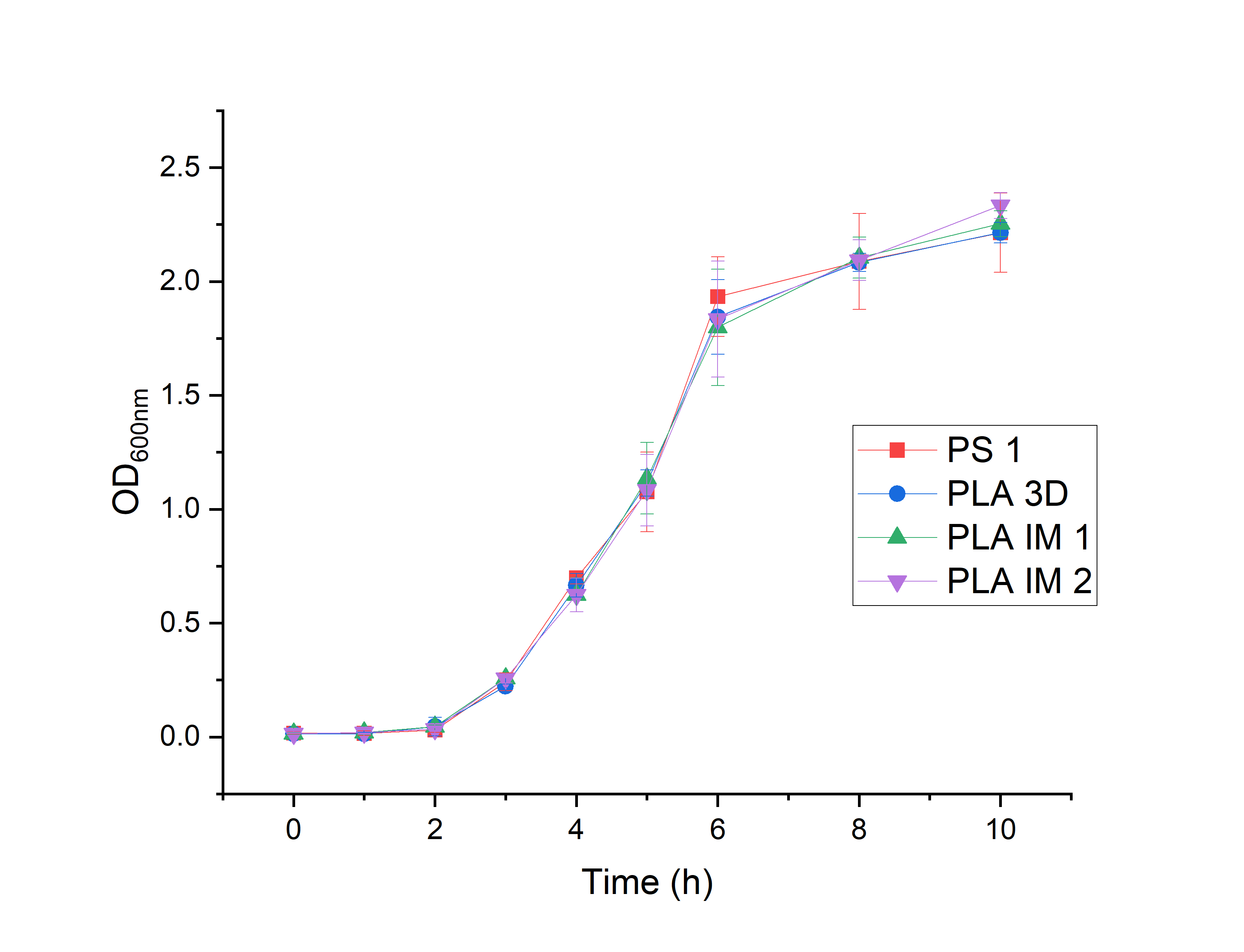

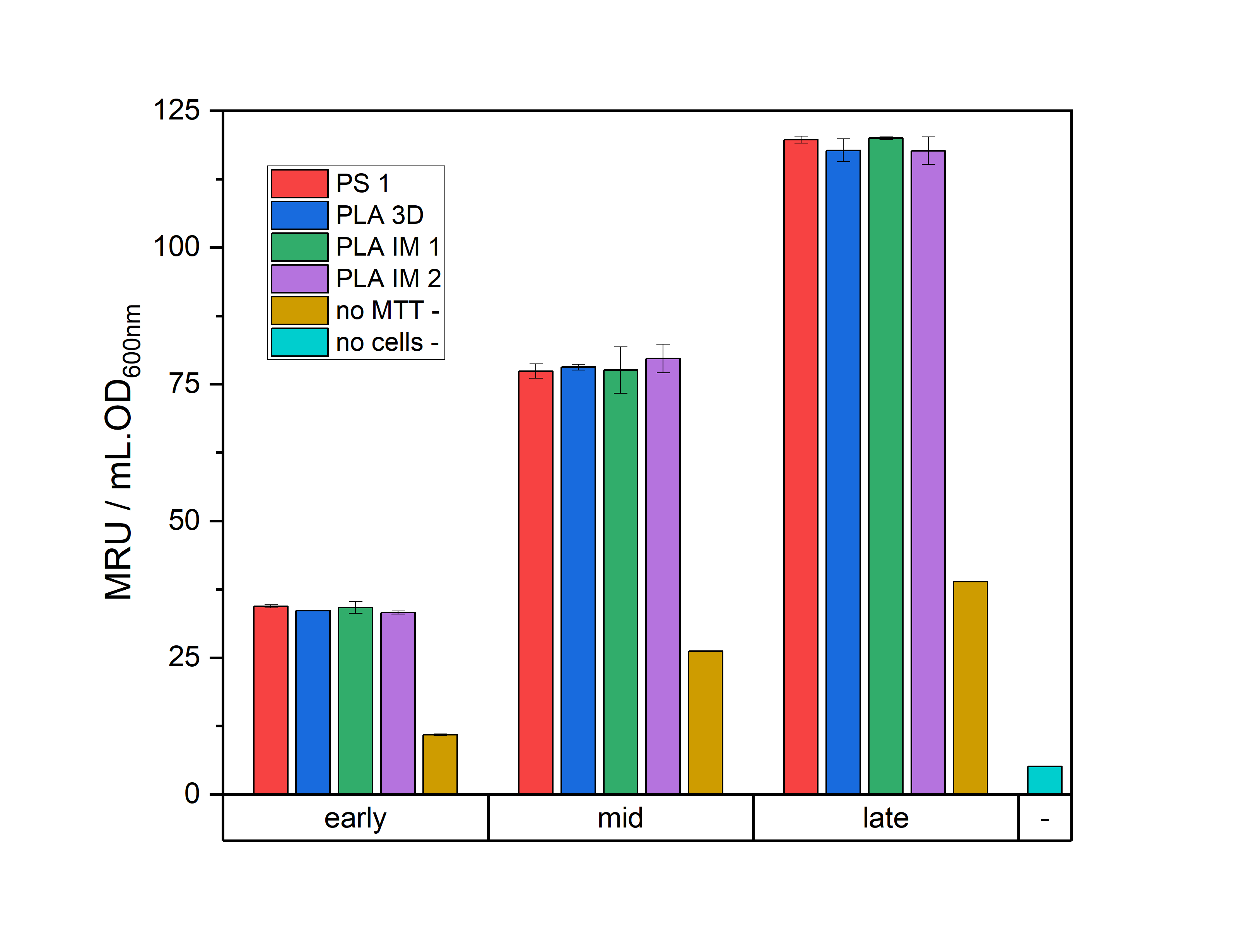


A

B

Figure S4. A) Growth curves of *E. coli* with starting colonies collected from indicated Petri dishes and grown in petrochemical PC conical flasks. The optical density at 600 nm was measured over 10 h, in LB medium at 37 °C. B) MRU values (MTT Reduction Units) of *E. coli* with starting colonies collected from indicated Petri dishes and grown in petrochemical PC conical flasks. Absorbance at 550 nm was measured when cells (early-, mid-, and late- exponential growth) were treated with MTT (0.45 g/L), incubated at 37 °C for 15 mins and resuspended in DMSO. Data is expressed as mean + SD (n = 3).


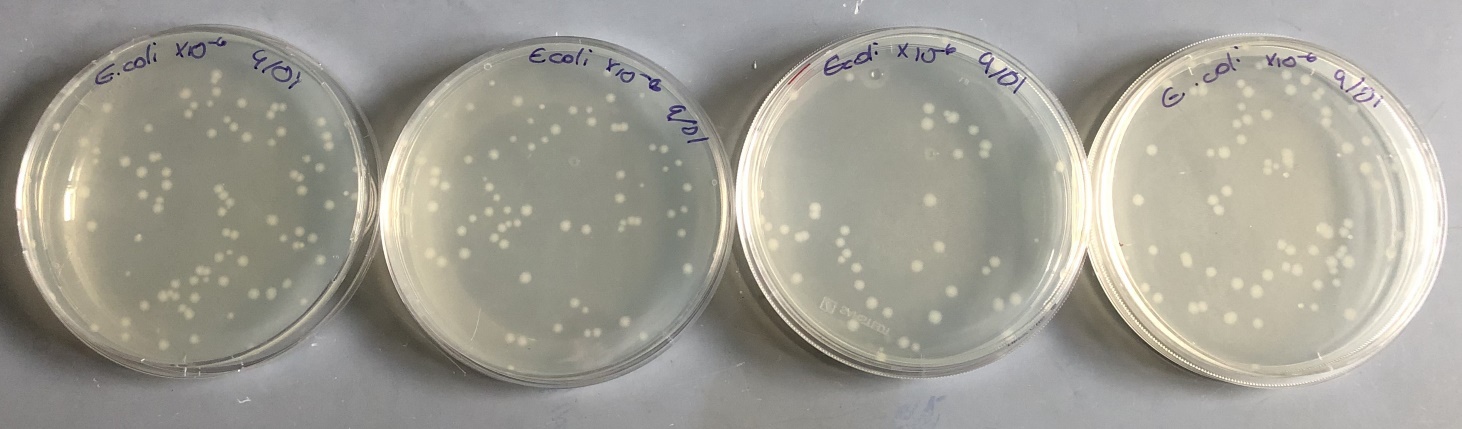


4

3

2

1

Figure S5. CFU counts of *E. coli* during late-exponential stage on 1) PS 1 2) PS 2 3) PS 3 4) PLA IM 2 Petri dishes.

Table S1. Indicating the time after inoculation of the overnight bacterial culture into fresh LB of the early-, mid-, and late-, exponential growth stages for each bacterial strain.

|  | Time after inoculation (h) | |
| --- | --- | --- |
|  | *E. coli* | *S. epidermidis* |
| Early- | 2 | 1 |
| Mid- | 3 | 2.5 |
| Late- | 5 | 4 |

Table S2. The CFU counts and average colony diameter of *E. coli* on the various Petri dishes under different conditions.

|  | PS 1 | PLA 3D printed | PLA IM 1 | PLA IM 2 | PS 2 | PS 3 |
| --- | --- | --- | --- | --- | --- | --- |
| CFU (mL·OD_600nm_) | | | | | | |
| early-exponential, 37°C, x10^-3^ | 2.89 + 0.02 x10^7^ | 2.08 + 0.06 x10^7^ | 1.65 + 0.20 x10^7^ | 1.85 + 0.12 x10^7^ | 2.34 + 0.02 x10^7^ | 2.50 + 0.2 x10^7^ |
| mid-exponential, 37°C x10^-5^ | 1.02 + 0.01 x10^8^ | 9.27 + 0.39 x10^7^ | 7.70 + 0.96 x10^7^ | 7.77 + 0.32 x10^7^ | 8.90 + 0.02 x10^7^ | 9.2 + 0.2 x10^7^ |
| late-exponential, 37°C, x10^-6^ | 2.99 + 0.02 x10^8^ | 2.33+ 0.11 x10^8^ | 1.60 + 0.28 x10^8^ | 1.71 + 0.37 x10^8^ | 2.34 + 0.02 x10^8^ | 2.52 + 0.2 x10^8^ |
| late-exponential, 37°C, x10^-7^ | 2.99 + 0.08 x10^8^ | - | - | 2.50 + 0.10 x10^8^ | 2.70 + 0.12 x10^8^ | - |
| late-exponential, 37°C, x10^-8^ | 2.91 + 0.10 x10^8^ | - | - | 2.43 + 0.12 x10^8^ | 2.43 + 0.13 x10^8^ | - |
| late-exponential, 30°C, x10^-6^ | 2.89 + 0.09 x10^8^ | - | - | 2.87 + 0.09 x10^8^ | 2.81 + 0.09 x10^8^ | - |
| late-exponential, 25°C, x10^-6^ | 2.99 + 0.21 x10^8^ | - | - | 2.71 + 0.20 x10^8^ | 2.74 + 0.24 x10^8^ | - |
| colony diameter (mm) | | | | | | |
| late-exponential, 37°C, x10^-6^ | 1.72 + 0.40 | - | - | 2.68 + 0.27 | 2.22 + 0.31 | - |
| late-exponential, 30°C, x10^-6^ | 1.92 + 0.65 | - | - | 2.01 + 0.69 | 1.99 + 0.70 | - |
| late-exponential, 25°C, x10^-6^ | 2.13 + 0.82 | - | - | 2.24 + 0.80 | 2.18 + 0.79 | - |

Table S3. Petri dish internal dimensions

| Petri dish | Diameter (cm) | Height (cm) |
| --- | --- | --- |
| PS 1 | 8.64 | 1.46 |
| PLA IM 2 | 8.58 | 1.57 |
